# Dominance of Orientation over Frequency in the Perception of 3-D Slant and Shape

**DOI:** 10.1371/journal.pone.0064958

**Published:** 2013-05-31

**Authors:** Danny M. Tam, Ji Shin, Andrea Li

**Affiliations:** 1 Dept of Psychology, City University of New York Graduate Center, New York, New York, United States of America; 2 Dept of Psychology, Queens College, Flushing, New York, United States of America; Université Paris 5, and CNRS, France

## Abstract

In images of textured three-dimensional surfaces, pattern changes can be characterized as changes in orientation and spatial frequency, features for which neurons in primary visual cortex are classically selective. Previously, we have demonstrated that correct 3-D shape perception is contingent on the visibility of orientation flows that run parallel to the surface curvature. We sought to determine the relative contributions of orientation modulations (OMs) and frequency modulations (FMs) for the detection of slant and shape from 3-D surfaces. Results show that 1) when OM and FM indicate inconsistent degrees of surface slant or curvature, observer responses were consistent with the slant or curvature specified by OM even if the FM indicated a slant or curvature in the opposite direction to the same degree. 2) For slanted surfaces, OM information dictates slant perception at both shallow and steep slants while FM information is effective only for steep slants. Together these results point to a dominant role of OM information in the perception of 3-D slant and shape.

## Introduction

The visual system is well able to extract information about 3-dimensional (3-D) shape from monocular cues. One potentially powerful monocular cue is the pattern or texture on a surface, which when projected in perspective, results in systematic changes in the texture in the image. These changes have commonly been referred to as texture gradients (e.g. size, density, compression) [1], and their abilities to convey 3-D shape have been studied extensively in the literature [2], [3], [4], [5], [6], [7]. Many gradient-based shape-from-texture models contain an assumption of homogeneity and thus use deviations from homogeneity to extract 3-D shape [8], [9], [10], [11], however not all surface textures are homogeneous. Since it is established that primary visual area V1 contains neurons sensitive to frequency and orientation, attempts to develop biologically plausible models of shape-from-texture have instead focused on how the cortex might decompose an image based on its local spatial frequency variations [12], [13], [14], [15], [16] and orientation components [17], [18], [19], [20], [21], [22]. It has since been demonstrated that correct 3-D shape perception hinges on the visibility of patterns of orientation flows formed by perspective convergence, irrespective of texture homogeneity [18], [19], [20], [21], [22]. Orientation flows play a critical role in 3-D shape perception not only in textured surfaces [23], [24] but also in specular and shaded surfaces [25], [26], [27], and thus provide a generic source of information for neural models of 3-D shape.

Recent adaptation studies have shown support for neural mechanisms that extract orientation flow information that exhibit invariance to the nature of the texture pattern [28], [29]. Physiological and imaging studies have begun to isolate neurons in extra-striate areas, such as the intra-parietal sulcus, that respond selectively to 3-D surface slant and curvature defined by texture and shading cues [30], [31], [32], [33]. However, little is understood about how the underlying neural mechanisms might extract orientation flow patterns. Additionally, although orientation flows appear to be critical for 3-D shape perception, it is unclear the extent to which they contribute to shape perception relative to other cues for 3-D shape and slant, such as changes in spatial frequency. The detectability of changes in frequency information (or frequency modulations) across a textured surface depends on the base frequency and the amount of change brought about by the surface slant [34] however, oriented higher spatial frequency components may not contain sufficient spectral energy to provide effective information for judging perceived slant [24]. Although frequency modulations and density gradients appear to convey surface slant for large fields of view [35], for smaller fields of view, frequency modulations in isolation can provide ambiguous information about the direction of surface slant and thus may not be considered a generally reliable cue to 3-D shape [18], [36], [20].

**Figure 1:**
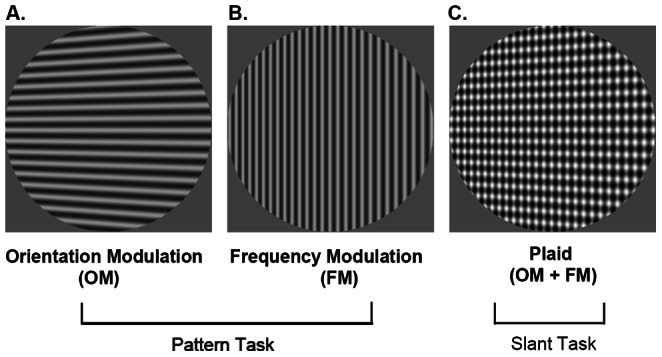
Texture-mapped developable planar surfaces used in Experiment 1. Surfaces shown here were slanted 30 deg to the left around a vertical axis of rotation. (A) Surfaces mapped with horizontal gratings predominantly produced changes in orientation (orientation modulations or OM). (B) Surfaces mapped with vertical gratings produced changes in frequency (frequency modulations or FM). (C) Surfaces mapped with plaid textures, which were the sum of horizontal and vertical gratings, contained both OM and FM changes.

The goal of the current study is to examine the contributions of changes in orientation and frequency to the detection of slant and shape from 3-D surfaces projected in perspective. Our hypothesis is that the visibility of orientation and frequency information in perspective images is directly linked to the perception of 3-D slant and shape. We used planar and corrugated surfaces texture-mapped with grating patterns. Planar surfaces were slanted out of the fronto-parallel plane, and corrugated surfaces varied in concavity/convexity. Examples of the planar stimuli are shown in Figure 1. Orientation modulations (OMs) are formed from gratings oriented perpendicular to the axis of slant. The contours of these gratings converge and diverge when the surface is rotated about the slant axis and viewed in perspective (Figure 1A). Frequency modulations (FMs) are formed from gratings oriented parallel to the axis of slant, which change in spatial frequency across the projected image (Figure 1B). In Experiment 1, we sought to determine the dominant cue to 3-D shape perception for surfaces in which the OMs and FMs specified inconsistent directions of slant and shape by quantifying whether the perception of slant or curvature was consistent with that specified by OMs or that specified by FMs. In Experiment 2, we sought to determine the extent to which these cues contribute to slant perception when specifying consistent slants by comparing the amount of surface slant required to detect pattern changes in OM and FM to the amount of surface slant required to detect slant for a plaid-textured surface containing both OM and FM changes.

## Methods

All research followed the tenets of the World Medical Association Declaration of Helsinki and informed written consent was obtained from the observers after explanation of the nature of the study. The research was approved by the Queens College Institutional Review Board.

### 1. Apparatus and Presentation

All stimuli were generated in MATLAB, converted to bitmap format in Adobe Photoshop CS5 and presented on a calibrated 22″ Mitsubishi Diamond Pro 2070 flat screen CRT monitor with a 1024×768 pixel resolution at a refresh rate of 100 Hz. The monitor was driven by a Cambridge Research Systems ViSaGe (CRS) Visual Stimulus Generator running on a 3.2 GHz Pentium 4 PC. Experimental code was written in MATLAB 7.4 using the CRS Toolbox. Observer responses were recorded using a CRS CB6 infrared response box.

Observers’ head positions were fixed with the use of a chinrest situated 1 m from the stimulus monitor. In all conditions, the center of the screen was level with the observer’s eye. When one stimulus was on screen, it was centered, or if two stimuli were presented simultaneously they were both equally spaced 3.25 deg to the left and right of center. Viewing was monocular in a dimly lit room, with no audio feedback except to indicate that a response had been registered.

### 2. Experiment 1: Perceived Slant and Curvature from Inconsistent Orientation and Frequency Cues

Li and Zaidi [19] demonstrated phenomenologically that for corrugated surfaces, OM information dominated when OMs and FMs specified inconsistent curvatures. In the first experiment, we sought to systematically quantify this for planar slanted and corrugated surfaces. We tested slanted and corrugated surfaces containing OM and FM information specifying slants and curvatures that were inconsistent by varying degrees, where the slant or curvature specified by one modulation was fixed, while the slant or curvature specified by the other was varied (and vice versa).

#### 2.1. Stimuli

All stimuli were presented in a circular aperture subtending 6.5 deg (11.36×11.36 cm) on a grey background with a mean luminance of 54 cd/m^2^. This field of view was chosen to allow for a consistent presentation of stimuli across both experiments. A black central fixation cross subtending 17×17 arc min was present at the center of the screen for the entire duration of all sessions.

**Figure 2:**
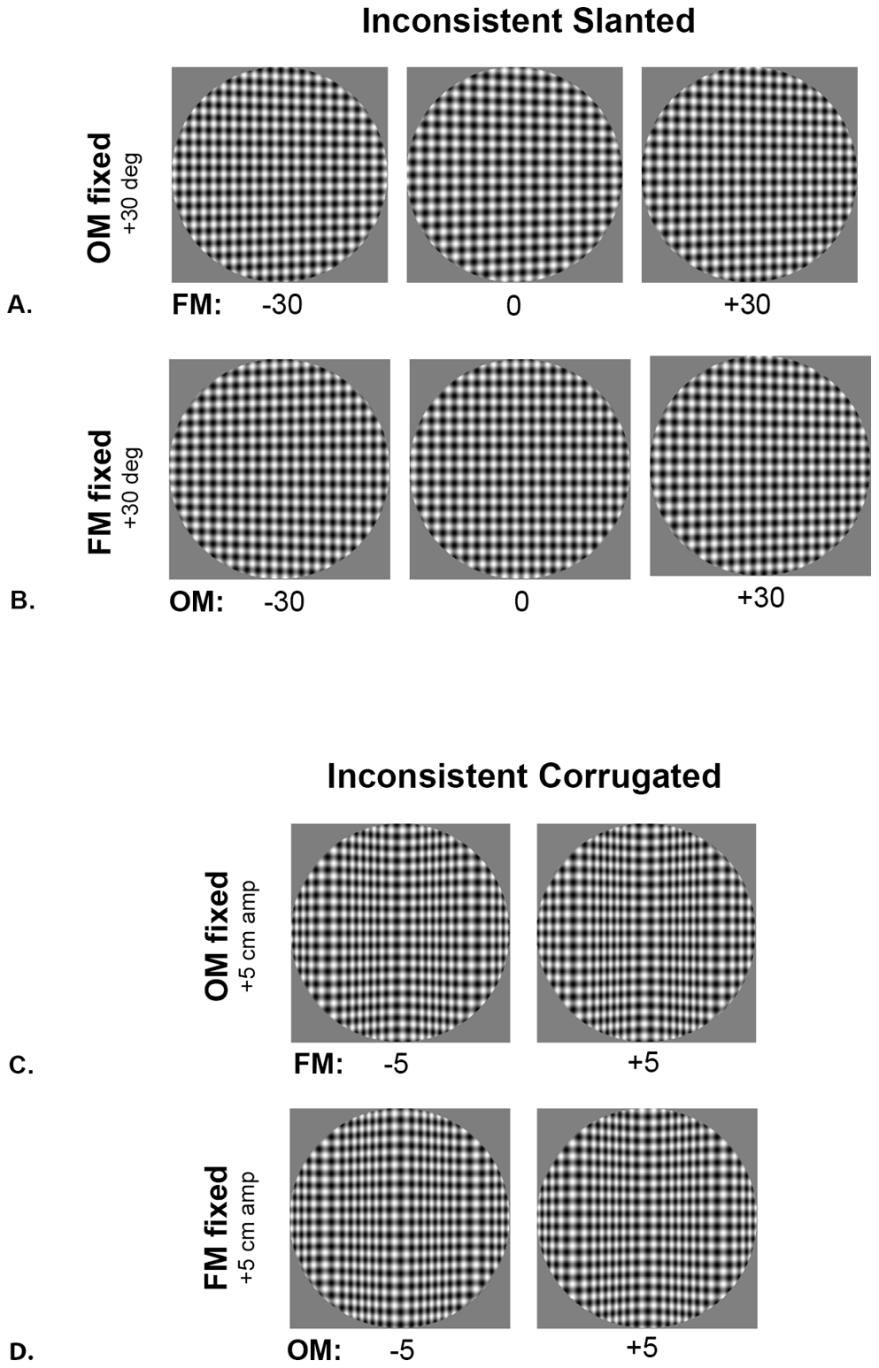
Slanted and corrugated surfaces containing inconsistent OM and FM information. Panels A and B illustrate planar surfaces mapped with a horizontal-vertical plaid containing both OM and FM information. (A) Slant specified by the OMs was fixed at ±30 deg while that specified by the FMs was varied from specifying the same slant angle to a slant of equal but opposite sign (B) Slant specified by the FMs fixed at ±30 deg while the slant angle specified by the OMs was similarly varied. In the inconsistent corrugated condition (C and D), stimuli were texture mapped surfaces which were corrugated sinusoidally in depth as a function of horizontal position. The amplitude of the central concavity or convexity of the shapes was varied in cm. Panels C and D are examples of our two stimuli set, where in one (C) the amplitude specified by the OMs was fixed at ±5 cm amp while that specified by the FMs was varied from specifying the same curvature to a curvature of equal but opposite sign, and the other (D) illustrates when amplitudes specified by the FMs were fixed at ±5 cm amp while that specified by the OMs was similarly varied.

In the *inconsistent slanted* condition, stimuli were generated by mapping developable planar surfaces with different textures, slanting them around a vertical axis of rotation and projecting them in perspective. Surfaces were patterned with 2 cpd horizontal and vertical gratings at 50% contrast obtainable on the display monitor, and 2 cpd horizontal-vertical plaids at 100% contrast. Slanting the surfaces about a vertical axis and viewing them in perspective produces systematic changes in orientation (orientation modulations or OM) in the horizontal gratings and systematic frequency modulations (FM) in the vertical gratings (see Figure 1A and 1B). For one set of the stimuli (OM-fixed), the OMs were fixed in pattern specifying ±30 deg slant angles while the FMs were varied from specifying the same slant angle as the OMs to an angle of equal but opposite sign (in 10 deg increments) (see Figure 2A). The other set (FM-fixed) contained FMs specifying a fixed ±30 deg slant angle while the slant angle specified by the OMs was similarly varied (Figure 2B).

In the *inconsistent corrugated* condition, stimuli were also generated with textures mapped onto a developable surface corrugated sinusoidally in depth as a function of horizontal position and viewed in perspective. Each stimulus image contained 1.5 cycles of the corrugation with either a central concavity or convexity. Peak-to-trough amplitudes of the corrugations were varied in centimeters. For OM-fixed stimuli, the corrugation amplitude specified by the OMs was fixed at ±5 cm while the corrugation amplitude specified by the FMs was varied from specifying the same curvature to a curvature of equal but opposite sign (in 1 cm increments) (see Figure 2C). For FM-fixed stimuli, the corrugation amplitude was specified by FMs fixed at ±5 cm while the corrugation specified by the OMs was varied (Figure 2D).

#### 2.2. Procedure

All sessions began with a 60 sec fixation period to a mean grey screen followed by a tone to signal the start of the trials. An audio beep coincided with the presentation of each test stimulus. Stimuli were presented for 250 msec each. Following each stimulus presentation, a 500 msec Gaussian noise mask was presented to minimize potential afterimages of the test stimuli. The screen then remained at the mean grey until the observer made a response. Each session lasted approximately 15–20 minutes.

In the *inconsistent slanted* condition, observers performed a single interval task in which they were asked to indicate the direction of slant for a planar surface textured with a plaid containing both OMs and FMs. Both OM-fixed and FM-fixed stimuli were randomly interleaved into one session. There were a total of 280 trials (4 fixed OM/FM modulations at ±30 deg ×7 slant angles × 10 trials).

The *inconsistent corrugated* condition was analogous in design to the above condition, in which observers were given a single interval task but asked to indicate the sign of surface curvature (convex or concave) for a plaid textured corrugated surface. As in the above condition, OM-fixed and FM-fixed stimuli were randomly interleaved into one session. There were a total of 440 trials (4 fixed OM/FM modulations at ±5 cm amplitude × 11 corrugation amplitudes × 10 trials).

### 3. Experiment 2: Contributions of Orientation and Frequency to Perception of Surface Slant

The goal of this second experiment was to quantify the contributions of orientation and frequency to the perception of slant when the two specify consistent slants. We first measured the minimum amount of slant required to perceive pattern changes in OM and FM-only stimuli. We then compared these thresholds to the minimum slant required to perceive the 3-D slant of a surface containing both OM and FM information.

#### 3.1. Design

We sought to measure the perception of OM and FM patterns and determine how perceptions of these patterns, which are 2-D in nature, relate to the perception of 3-D surface slant. Thus there were two different types of tasks – tasks that required judgments about 2-D patterns and tasks that required judgments about 3-D surface slant. We examined pattern and slant percepts for two surface conditions: in the 0-deg condition, percepts were measured for surfaces around the fronto-parallel plane; in the 30-deg condition, percepts were measured for surfaces deviating in slant around an existing ±30 deg slant.

In the 0-deg condition, observers were presented with stimuli varying in slant around the fronto-parallel plane patterned with OMs, FMs, or both in a plaid (Figure 1). Stimuli were presented in a single interval paradigm and observers were asked to indicate the direction (left or right) in which the 2-D pattern changed (for OMs and FMs) or in which the surface appeared slanted in depth (plaid). Specifically, for OMs observers were asked to indicate the direction in which the lines converged, and for the FMs the direction in which the width of the bars decreased. Each of the three pattern types was tested in a single session consisting of 270 trials (18 slant angles × 15 trials) in which the stimuli were presented in random order. Each observer ran 3 sessions for each pattern type for a total of 9 sessions in the experiment. The order in which the stimuli conditions were run was randomized within and across observers.

In the 30-deg condition, observers were presented with stimuli varying in slant around an existing ±30 deg slant also patterned with OMs, FMs, or a plaid. In each trial, two stimuli were presented simultaneously to the left and right of fixation; one stimulus was a base slant set at a ±30 deg slant, while the other was a test slant that deviated from the base slant and was always more steeply slanted than the base slant. Observers were required to indicate the stimulus that contained a greater change in OM or FM pattern, or that appeared more slanted in depth. There were 210 trials in each session for the (7 slant angles × 15 trials × 2 fixed base slants). The test slant presented as well as the side on which the base and test slant were presented onscreen were randomized from trial to trial.

The motivation for using a single interval task for the 0-deg condition was to ensure that observers judged the FM gradient rather than the overall frequency or effective contrast of the stimulus. As surface slant is varied, so does the average frequency and effective contrast of the FM stimuli, and these changes are more visible for surfaces varying around the fronto-parallel plane over the range of slants required to determine thresholds. Thus, in a two-interval paradigm around fronto-parallel, it would be possible for observers to use average frequency or contrast as a cue. Although these potential confounds still exist for the 30-deg condition, the range of slants required to detect pattern changes in the FM stimuli (and therefore the range of average frequencies across these stimuli) was greatly reduced compared to the 0-deg condition, and an additional control experiment described in the Results section indicated observers were not using effective contrast as a cue in this condition.

Although our instructions to observers were specifically worded in the context of judging ‘pattern’ (2-D) vs. ‘slant’ (3-D), the response choices were identical across the two surface conditions (choose left vs. right in the 0-deg condition, choose the left or right stimulus in the 30-deg conditions). Thus, it is difficult to ensure that observers were specifically making 2-D vs. 3-D judgments, e.g. observers might have been indicating the direction of line convergence when asked to make judgments about surface slant, and/or when asked to make judgments about the direction of line convergence, they could have been indicating the direction of perceived slant. Two things should be noted here: 1) As may be illustrated in Figure 1, the plaid stimuli appear more planar than OM and FM stimuli, and our naïve observers in fact described the OM and FM stimuli as not being very ’surfacey’. Thus observers indicated that making a judgment about the 3-D slant of the plaid surface in depth was not difficult and felt intuitive. Since OMs and FMs in isolation appeared to convey minimal information about surface depth, making judgments about the direction in which the lines converged or in which the bar width decreased also was not difficult, and more intuitive than judging the direction of the slant of the surface for these stimuli. We speculate that making judgments about surface slant with OM and FM stimuli would have in fact been more difficult than making judgments about pattern changes within each, especially in the case of the FM stimuli. 2) Although other paradigms exist to measure the absolute slant or shape percept of a surface (e.g. changing a depth gauge on a surface to match its slant, or matching the slant of an aerially viewed line to match the perceived slant of a surface), these paradigms would not directly provide the type of data we required in this experiment. Specifically, we sought to determine the minimum slant angles required to perceive pattern changes (OMs and FMs) and compare them to the minimum slant angles required to perceive deviations of surface slant (plaid). The goal of the experiment was to be able to compare these conditions using a single metric of slant angle.

#### 3.2. Stimuli

Three main sets of test stimuli were generated by mapping developable planar surfaces with different textures, slanting them around a vertical axis of rotation and projecting them in perspective. For the OM and FM stimuli, surfaces were mapped with 2 cpd horizontal or vertical gratings at 50% contrast obtainable on the monitor (Figure 1A and 1B). For the plaid stimuli, the horizontal and vertical gratings were summed to generate plaid textured surfaces at 100% contrast obtainable on the monitor, containing both OMs and FMs when viewed in perspective (Figure 1C). The size of the stimuli and the manner in which they were displayed and viewed by observers were the same as those in Experiment 1.

A preliminary study was run to determine the appropriate range of slants for each condition and surface texture to be used in a method of constant stimuli. For each of the OM, FM, and plaid patterns in the 0-deg condition, 18 test stimuli were generated by slanting the patterns around a vertical axis of rotation, with half the stimuli slanted leftward and the other half slanted rightward. Slant angles of the OM stimuli were ±1, 2, 4, 6, 8, 10, 12, 14 and 16 deg; slant angles of the FM stimuli were ±1, 8, 16, 24, 32, 40, 48, 56, and 64 deg; and slant angles of the plaid textured surfaces were ±1, 3, 6, 9, 12, 15, 18, 21 and 24 deg.

For each of the OM, FM, and plaid patterns in the 30-deg condition, 14 test stimuli were generated deviating from a fixed base slant of ±30 deg. For both the OM stimuli and the plaid surfaces, the stimuli were +2, 4, 6, 8, 10, 12, and 14 deg deviating from the fixed base slant of ±30. For the FM stimuli, the slant angles were ±1, 2, 3, 4, 5, 6, 7 deg deviating from the fixed base slant of ±30.

For generality, the 0- and 30-deg conditions were repeated using stimuli that were rotated about a horizontal axis (i.e. floor/ceiling slant).

#### 3.3. Procedure

All sessions began with a 60 sec fixation period to a mean grey screen followed by a tone to signal the start of the trials. An audio beep coincided with the presentation of each test stimulus. Following each stimulus presentation, there was a 500 msec Gaussian noise mask. The screen then remained at the mean grey until the observer made a response. Each session lasted approximately 15–20 minutes.

In the 0-deg condition, stimuli were each presented for 250 msec. For the OM and FM patterns, observers were asked to indicate the direction of convergence in OMs for the horizontal gratings, or the direction of bar width gradient decrease for the FMs in vertical gratings. For plaid stimuli, observers were asked to indicate the direction of surface slant for a horizontal-vertical plaid.

In the 30-deg condition, stimuli were presented for 800 msec, with the extra time being necessary as observers were being asked to discriminate between two stimuli presented side by side. Observers were asked to indicate which of the two stimuli contained either the greater degree of OM or FM change, or the greater degree of surface slant in the plaid surface.

### 4. Observers

Three of the authors and 2 naïve observers participated in both experiments. All had normal or corrected-to-normal visual acuity.

## Results

### 1. Experiment 1: Perceived Slant and Curvature from Inconsistent Orientation and Frequency Cues

**Figure 3:**
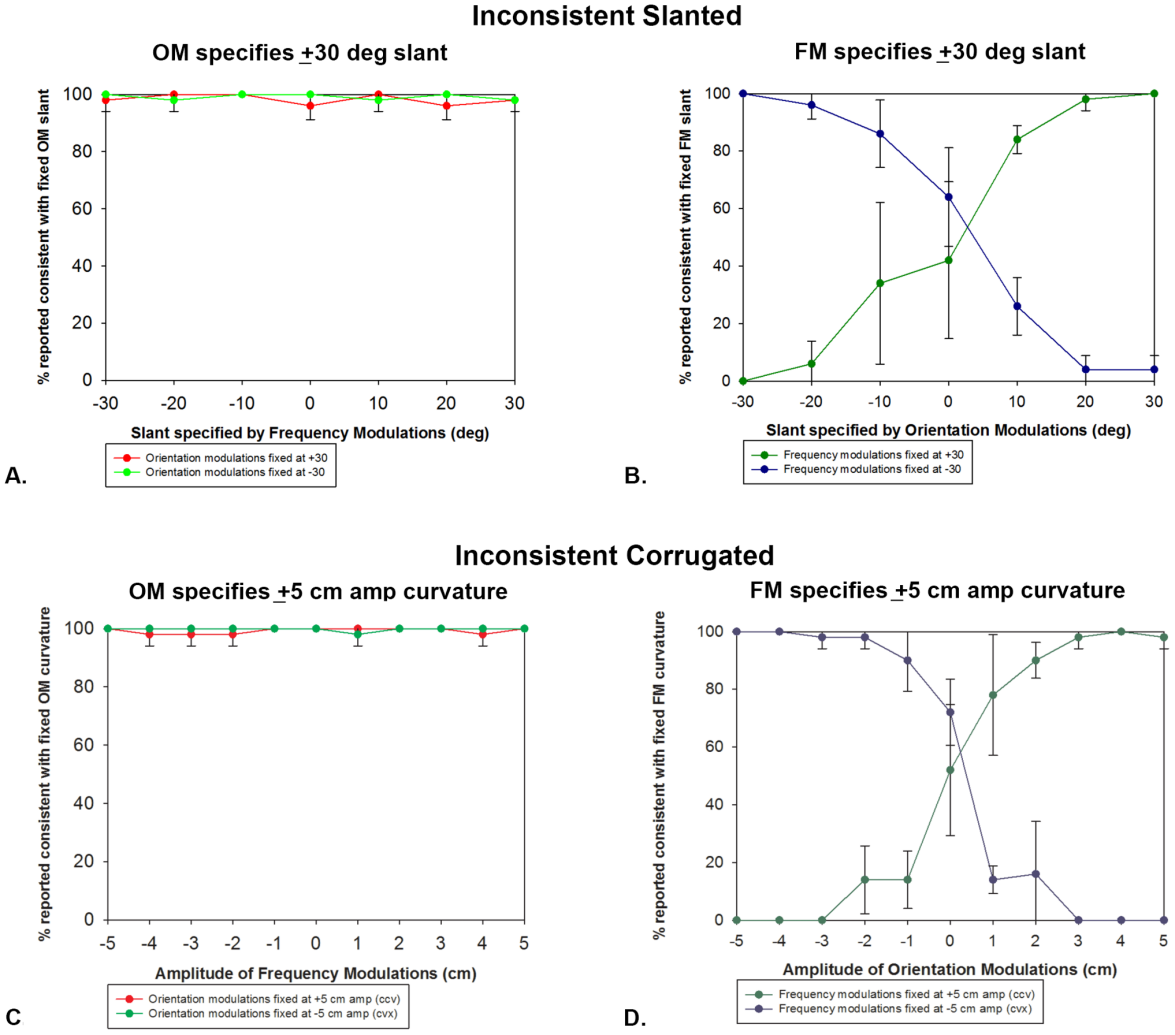
Perceived direction of slant and curvature for surfaces with inconsistent OM and FM information. Percentage of trials reported consistent with a pattern at a fixed slant angle of ±30 deg (top) or corrugation amplitude of ±5 cm (bottom) plotted vs. the range of slants/corrugations specified by the other pattern. For each panel, data are averaged across five observers and error bars represent 95% confidence intervals.

For the inconsistent slanted condition, the percentage of trials reported consistent with the pattern (OM or FM) at a fixed base slant of ±30 deg was plotted vs. the range of slants specified by the other pattern (Figure 3A and 3B). Data for the trials containing the fixed pattern at positive and negative base slants were plotted separately in each graph. Significance was determined using 95% confidence intervals, which were plotted as the error bars in all graphs. When the slant specified by the OMs was fixed at ±30 deg, responses for the direction of slant were consistent with the OMs, even if the FMs specified a slant of the opposite sign to the same degree (Figure 3A). In contrast, when the slant specified by the FMs was fixed, the proportion of responses consistent with FM-specified slant was greatest only when the FMs and OMs specified the same slant (Figure 3B). With increasing deviations of slant specified by the OMs, the percentage of responses consistent with OM-specified slant increased such that they were completely consistent with this slant when the OMs specified a slant to the same degree as the fixed FMs but of opposite sign.

The same pattern of results was observed for the inconsistent corrugated condition. Figure 3C and 3D plot the percentage of trials reported consistent with the pattern (OM or FM) at a fixed corrugation amplitude of ±5 cm vs. the range of amplitudes specified by the other pattern. As was the case for inconsistent slanted surfaces, observer responses followed the corrugation specified by the OMs, regardless of whether the FMs specified a concavity or convexity. Taken together, these results indicate that slant and shape judgments were dictated by OM information, even when the FMs specified a slant or shape in the opposite direction to the same degree.

### 2. Experiment 2: Contributions of Orientation and Frequency to Perception of Surface Slant

**Figure 4:**
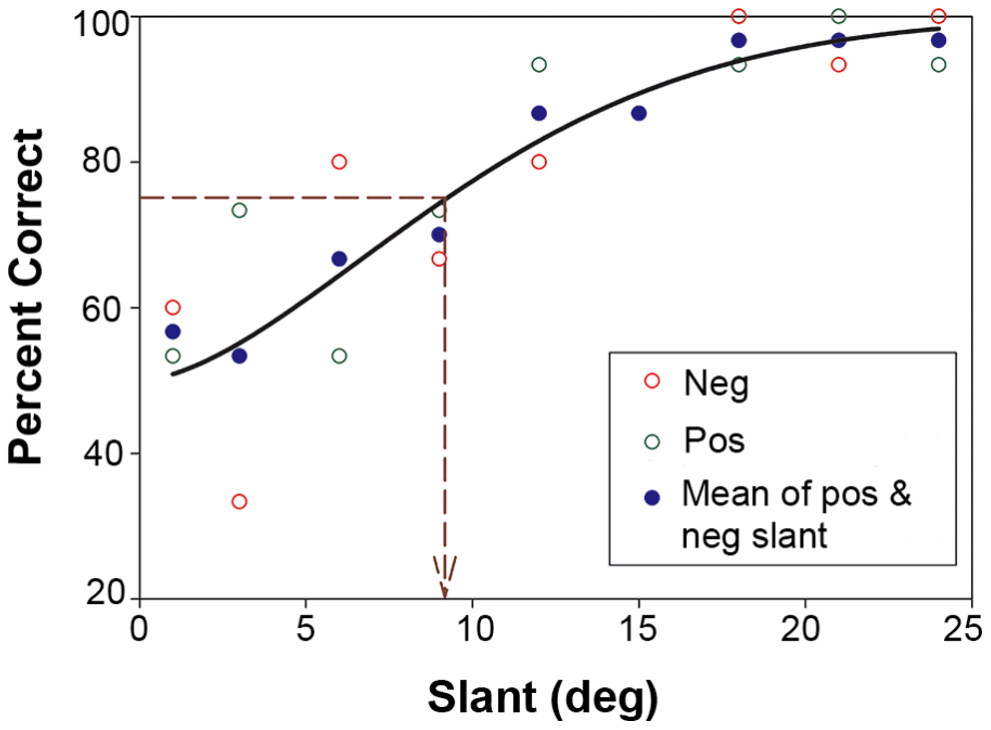
Sample data from a session using plaid stimuli in the 30-deg condition for one observer. Figure plots the percentage of correct trials indicating which of two stimuli presented had the greater surface slant vs. the degree of surface slant in degrees. Data points for positive and negative slant trials were averaged together and fitted with a Weibull function to extrapolate the threshold. The detection threshold was quantified as the minimum amount of surface slant needed for an observer to correctly identify the direction of pattern change or surface slant 75% of the time.

For each of the three patterns in the 0-deg condition, the percentage of correct trials (i.e. reported consistent with the simulated direction of pattern (OM/FM) change or surface slant) was plotted vs. the degree of surface slant. For the 30-deg condition, the percentage of correct trials indicating which of two stimuli presented had the greater pattern change or surface slant was plotted against the degree of surface slant from the fixed base slant. The percent correct for positive and negative slants were averaged together at each slant value, with the threshold extrapolated from a Weibull fit of the averaged data. As an example, Figure 4 depicts the data for one observer for one pattern in the 0-deg condition. The threshold was quantified from the fitted averaged data as the minimum amount of surface slant needed for an observer to correctly identify the direction of pattern change or surface slant 75% of the time.

**Figure 5:**
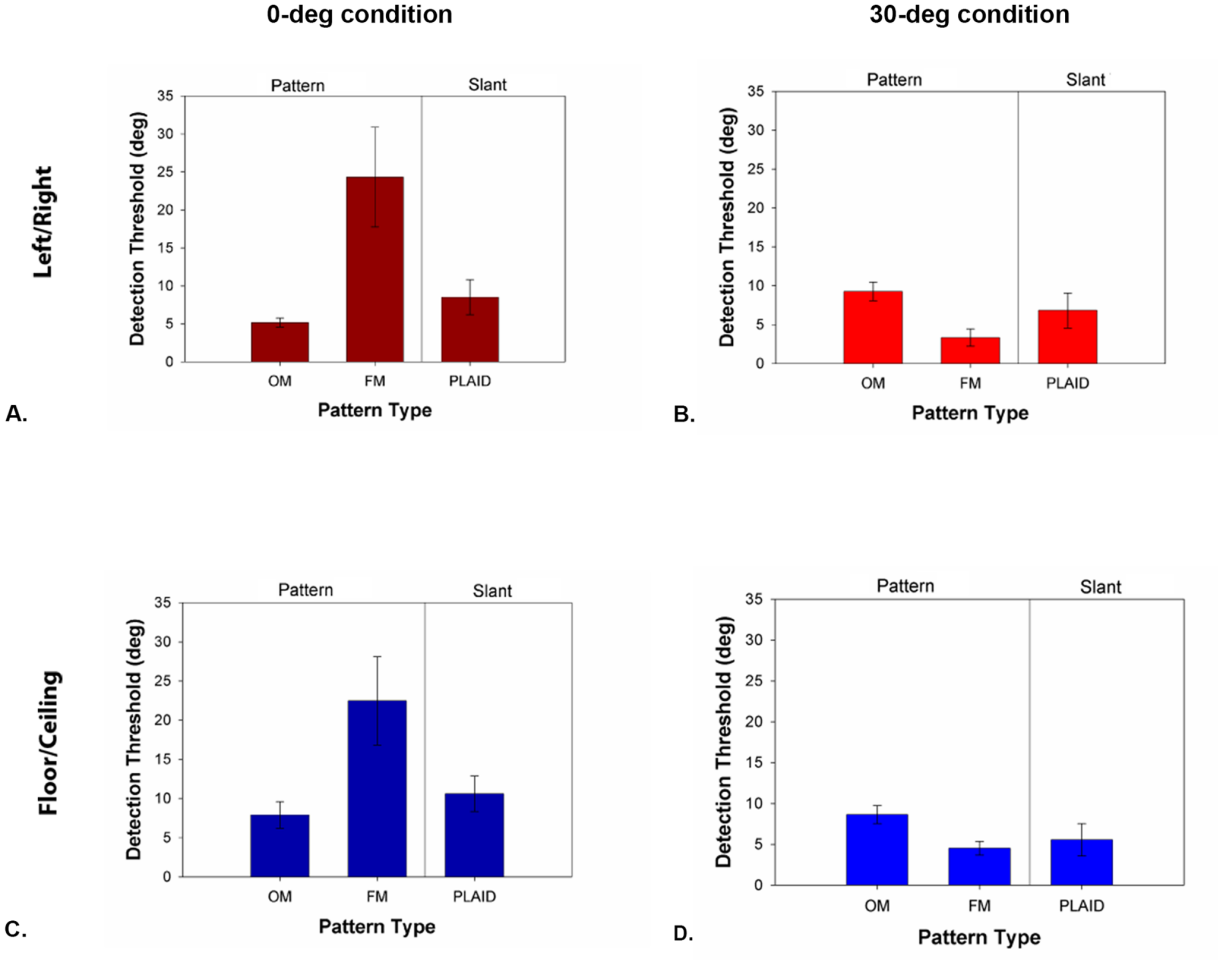
Contributions of orientation and frequency to the perception of shallow and steep slants. Detection thresholds in degrees for the three stimuli types: OM, FM, and plaid, for both the 0-deg (left) and 30-deg condition (right). Panels A and B are data for stimuli slanted about a vertical axis of rotation, and panels C and D for stimuli slanted about a horizontal axis of rotation. For the 30-deg condition, the detection threshold represented the amount of slant deviating from a fixed base slant of ±30 deg. For each panel, data are averaged across five observers and error bars represent 95% confidence intervals.

Data averaged across the five observers are shown in Figure 5. Each panel plots the thresholds in degrees for the three pattern types for both the 0-deg (Figure 5A) and 30-deg (Figure 5B) conditions. Figure 5C and 5D show results for the conditions when stimuli were rotated about a horizontal axis (i.e. floor/ceiling slant). Significance was determined using 95% confidence intervals, which are plotted as error bars in all graphs. For the 30-deg conditions (Figure 5B and 5D), the threshold represented the amount of slant deviating from a fixed base slant of ±30 deg.

In the 0-deg condition, the highest slant thresholds were consistently observed for the FM pattern tasks regardless of axis of rotation. No significant difference was observed between the amount of surface slant needed to identify the direction of pattern change for OMs and that needed to detect the surface slant of the plaid. In the 30-deg condition, thresholds for determining which of two stimuli had greater OM change was consistent with that needed to judge surface slant of the plaid. Interestingly, the thresholds for FM stimuli in the 30-deg conditions were significantly lower than that of the OMs irrespective of axis of rotation. For surfaces mapped with a plaid texture that were rotated around a vertical axis, there was no significant difference in the amount of surface slant required for observers to detect the direction of surface slant in the 0-deg condition and that needed to discriminate which of two stimuli contained a greater degree of surface slant in the 30-deg condition. However, for surfaces rotated around a horizontal axis (i.e., floor/ceiling slant), the amount of surface slant needed to detect slant around fronto-parallel was significantly greater than that around 30-deg. These results indicate that slant perception appears to be dictated by the detectability of OMs for slants around the fronto-parallel plane. At steeper slants, OMs and FMs appear to be similarly effective at conveying surface slant.

As the effective contrast of the FM stimuli decreased with increasing slant, one concern was that observers may have been utilizing differences in effective contrast as a cue rather than perceived FM gradients. An additional experiment was conducted using the method of limits in which observers were presented with an FM pattern at 50% contrast at the maximum slant for these stimuli used in the 30-deg condition (±37 deg). These were presented together with FM stimuli of the different slants used in the 30-deg condition (in the same spatial configuration as that used in that condition), in which the contrast was varied in ascending and descending series for each slant. Observers were asked to indicate which of the two stimuli was higher in contrast, and thresholds for equated contrast were estimated from 6 series of trials (3 ascending and 3 descending) for each slant. The contrast level of all the FM stimuli in the 30-deg condition which observers judged as perceptually similar to the contrast of the FM pattern at the maximum slant was not significantly different. Thus we do not think that effective contrast was a confounding factor in our FM stimuli.

## Discussion

Our results show that when the OMs and FMs on a slanted or corrugated surface specify inconsistent directions of slant or curvature, the percept is dictated by the OMs. In addition, slant perception is dictated by the detectability of OMs for slants around the fronto-parallel plane, and both OMs and FMs are similarly effective at conveying surface slant around steeper slants. For surfaces around fronto-parallel, an increase in surface slant organically results in changes in orientation that are more readily visible than changes in frequency. The amount of surface slant needed to detect changes in slant was therefore more consistent with that needed to detect a pattern change for OMs than FMs. For steeper slants, the amount of slant needed to detect FMs was actually less than that needed to detect orientation changes. The marked drop in FM thresholds with increasing slant suggests that for slanted surfaces, a small amount of slant results in more visible changes in FMs relative to OMs. There is likely a combination of mechanisms responsible for extracting both OMs and FMs for the perception of slanted surfaces, but our results do not distinguish their specific contributions at steeper slants.

Use of the plaid stimulus introduces an additional, potentially useful cue of orthogonality whereby deviations from orthogonality of the horizontal and vertical plaid in the image increase with surface slant [37]. Given that these deviations are correlated with the orientation modulations (greater deviations occurring where orientation modulations are greater), one consideration is that they may provide an apparent bias for the orientation modulations as a cue for surface slant. Deviations from orthogonality are also correlated with frequency modulations (deviations from orthogonality increasing with increasing local frequency), although the magnitude of the frequency modulations does not affect the magnitude of the deviations from orthogonality which depend on local orientation. The question thus is whether the visual system is using orthogonality to make judgments about surface slant in Experiment 1. Our experiment is unfortunately not designed to determine whether or not this is the case. However, since the magnitude of local orthogonality depends on the magnitude of the local orientation modulation, it is still clear that the visual system is using orientation information to perceive the surface slant, regardless of whether orthogonality is also being used.

Interestingly, for surfaces around fronto-parallel in the 0-deg condition, slant thresholds for the plaid surfaces were higher than pattern thresholds for the OMs across all observers, although this trend was not significant when averaged across observers. Previous physiological work has shown that striate neurons in cat and primates have a decreased response if a stimulus at the preferred orientation is superimposed by an orthogonal stimulus, a process known as cross-orientation suppression [38], [39]. It is possible that the addition of the orthogonally oriented frequency components act to suppress the visibility of the OMs, thus the observed trend across observers of lower thresholds for OM stimuli may reflect previous findings that show release from cross-orientation suppression increases the visibility of the critical orientation information which facilitates decoding of 3-D shapes [40]. However, for surfaces that are already substantially slanted in the 30-deg condition, any release from cross-orientation suppression does not seem to be evident: pattern thresholds for OMs and slant thresholds for the plaid were similar, suggesting that the presence of the FM had no effect on the perception of the OMs and therefore perceived surface slant. This may be due to the fact that at steeper slants, the difference in FMs between the horizontal and vertical grating components is large, and thus the frequency-selective cross-orientation suppression isolated in Li and Zaidi [40] may not be at play.

Previously we have shown that in isolation, FMs resulting from developable surface texture mappings, such as the planar and corrugated surfaces used in this study, can lead to misperceptions of surface shape, specifically concave surfaces tend to appear convex [18], [36], [20]. These misperceptions result because FMs are interpreted as cues to distance between the viewer and the surface rather than as cues to surface slant. For texture mappings in which FMs consistently reflect distance, correct surface shape is perceived [20]. Thus how FMs are perceived depends on the type of texture mapping used. In contrast, similar patterns of OMs appear to arise across many different types of texture mappings and consistently provide correct shape information. Therefore, we have considered them more reliable sources of information to 3-D shape. The results presented here further bolster our previous findings by showing that OMs provide consistent cues to surface slant at shallow and steep slants, whereas FMs are useful only around steeper slants. It is worth noting that the OM stimuli used in our study contain minimal modulations in frequency: in fact it is not possible to generate texture-mapped stimuli that contain OMs in the total absence of FMs without additional image processing of the projected image. Thus if one were interested in an independent analysis of OMs vs. FMs (e.g. in any sort of cue combination paradigm), the stimuli used in our study would not be appropriate. It is also worth noting that Todd et al. [35] showed that FMs can convey surface slant for steep slants ranging up to 65 deg, and that increasing the optical window improves these slant judgments. Although we did not test the effect of varying field of views on slant judgments, our findings are consistent with their finding that steeper slants elicit contributions from FMs in the judgment of surface slant. Since our FM pattern thresholds were similar in magnitude to the slant thresholds for steeper slants, it seems likely that FMs are in fact contributing to slant judgments, even at our slant values which were quite a bit shallower, and our field of view that was smaller, than those used in Todd et al. [35].

Our results suggest that the perception of 3-D slant and shape from texture must involve the neural extraction of OMs (at shallow and steep slants) and FMs (at steep slants). Results from adaptation studies suggest that mechanisms that extract OMs are invariant to how the OMs are defined over a range of spatial frequencies and thus inevitably lie in extra-striate visual areas [28], [29]. The nature of the neural mechanisms that might extract FMs, and the mechanisms that combine the two sources of information to yield the 3-D shape percept have yet to be explored.
